# The impact of writing on academic performance for medical students

**DOI:** 10.1186/s12909-021-02485-2

**Published:** 2021-01-19

**Authors:** Songeui Kim, Ji Won Yang, Jaeseo Lim, Seunghee Lee, Jungjoon Ihm, Jooyong Park

**Affiliations:** 1 Department of Psychology, Seoul National University, Seoul, South Korea; 2 Interdisciplinary Program in Cognitive Science, Seoul National University, Seoul, South Korea; 3 Department of Medical Education, College of Medicine, Seoul National University, Seoul, South Korea; 4 Dental Research Institute, School of Dentistry, Seoul National University, Seoul, South Korea

**Keywords:** Academic performance, Effects of writing, Higher-order thinking, Medical education

## Abstract

**Background:**

Writing is a useful learning activity that promotes higher-order thinking, but there are limited studies that prove its effectiveness. In previous research, researchers tested the effect of summary writing on students’ comprehension and found no significant difference from that of re-studying texts. The purpose of this study, therefore, is to expand previous findings and investigate the effect of two types of writing tasks on medical students’ academic performance, specifically in the transfer of knowledge.

**Methods:**

An experiment was conducted with 139 medical students from Seoul National University College of Medicine. They were randomly assigned to three study conditions: self-study (SS), expository writing (EW), and argumentative writing (AW) group. Each group studied the given material by the method they were assigned, and they were tested on their comprehension and transfer of knowledge using rote-memory type items and transfer type items respectively.

**Results:**

The results showed that the two writing groups displayed better performance than the SS group in transfer type items, while there was no difference in scores between the EW and AW group. However, the three groups showed no significant difference in their scores for rote-memory type items. Also, there was a positive correlation between the writing scores and transfer type item scores in the AW group.

**Conclusions:**

This study provides empirical evidence for writing to be adopted in medical education for greater educational benefits. Our findings indicate that writing can enhance learning and higher-order thinking, which are critical for medical students.

## Background

“Writing organizes and clarifies our thoughts. Writing is how we think our way into a subject and make it our own. Writing enables us to find out what we know—and what we don’t know—about whatever we’re trying to learn.” [1] As Zinsser once stated, we can clarify what we know and what we do not know through writing. The process of writing requires writers to have a clear understanding of the subject matter [2] and make use of cognitive abilities. Specifically, writing helps students develop higher-order thinking skills that involve three cognitive processes - analysis, evaluation, and creation [3]. At the beginning of the writing process, students should first create a sound argument and express it clearly. Afterward, they should constantly analyze and evaluate what has been written to make a persuasive claim without logical fallacies.

These higher-order thinking skills are needed for medical students to grow as successful medical professionals [4]. Doctors need to diagnose the problem of a patient, mentally represent the situation, plan appropriate treatments, and evaluate the whole process to check against other possibilities [5–7]. With limited research regarding the learning effects of writing in medical education, some medical schools make use of writing in their classrooms. By way of illustration, students of Maastricht College of Medicine are required to submit a portfolio that includes reflection papers on the roles and abilities of medical professionals, scientists, and health care providers, respectively [8]. Since these essays are written multiple times over semesters or years, students have the opportunity to reflect upon their whole learning process and look back on what they have learned.

While writing should be emphasized to foster higher-order thinking skills for future medical professionals, previous literature reports conflicting evidence on the effect of writing [9–11]. Representatively, Spirgel and Delaney (2016) concluded that summary writing was not effective than re-studying learning materials [12]. They also found that students better remembered only the items that were included in the summary compared to those that were omitted. However, it is early to conclude that students cannot learn enough from practicing writing, because in the previous research, they only measured students’ comprehension using rote-memory test items without testing the transfer of knowledge. In the end, applying learned knowledge to different areas is as important as understanding difficult concepts to deal with new patients and novel situations every day. When learners apply information, strategies, and skills they have learned to new contexts, transfer of knowledge occurs [13]. In this context, while summary writing may not have been more effective in memorizing learned contents [9], there is evidence that writing facilitates the transfer of knowledge. For example, Boscolo and Mason (2001) found that learning historical events through writing could be transferred to learning concepts in a different domain, science [14]. Considering that the ultimate goal of education should be to improve thinking skills and application of knowledge, the effects of writing on learning should also be measured in the context of the transfer of knowledge along with accurate memorization [15, 16].

Besides, different types of writing could have different effects on students’ learning because each writing task focuses on different skill sets. For instance, summary writing requires skills such as classification, comparison, definition, and illustration; it requires a structured interpretation of the texts and thus can be more effective in improving comprehension [17]. On the other hand, through argumentation, students can engage in a deeper and more mature level of learning [18]. When writing an argumentative essay writers need to establish contested claims while formulating an explanation, generating counterarguments, and assessing them to support their own opinions [3]. While both types of writing can help students develop thinking skills, writing an argumentative essay involves more reasoning [19] and promotes critical thinking, like analyzing and synthesizing an argument [20]. All in all, depending on which type of task teachers use, writing can be more effective in stimulating the transfer of knowledge and higher-order thinking beyond comprehension.

Thus, in this study, we investigated the effects of writing on the transfer of knowledge in a medical education setting. If there is empirical evidence that writing can help learn factual knowledge and enhance higher-order thinking skills at the same time, writing can then be used to complement traditional methods of learning to foster medical professionals who have broader perspectives. We developed the following study design: medical students were first divided into three studying conditions, those who study by themselves (self-study: SS), those who study by writing a summary text (summary writing: SW), and those who study by writing an argumentative essay (argumentative writing: AW). Then, we measured students’ academic performance through a final test that assessed both rote-memory and transfer of knowledge. Our hypotheses are as follows: First, all groups (SS, SW, and AW) will show no significant difference in their performance in rote-memory type items. Second, students in the writing groups (SW and AW) will score higher in transfer-type items; specifically, students who study by writing an argumentative essay (AW) will perform better than those who write a summary text (SW).

## Methods

### Participants

Participants were recruited at the Seoul National University College of Medicine. Among 139 individuals, 48 were female. Twenty-three participants who failed to follow the directions were excluded from the study. Therefore, only the data from the remaining 116 participants were analyzed (*M*_*age*_ = 19.22, *SD*_*age*_ = 0.79).

### Material

The participants were instructed to study 4-page-long written material. The study methods varied depending on their assigned groups. The subject matter dealt with the relationship between youth’s cognitive development and musical skills. This subject was chosen because it is less likely to be affected by background knowledge since related courses are not provided to the medical school students. Also, it was convenient to devise final test questions and the writing tasks based on the topic as the material covered diverse concepts and theories. We were also able to refer to an already existing set of test questions on this topic, verified and used in the National Teacher Certification Examination in Korea.

### Experiment procedure

Participants were randomly assigned to either of the three groups: the SS (self-study) group, the SW (summary writing) group, and the AW (argumentative writing) group. For the SS group, participants were instructed to study the written material by themselves for 25 min. For the writing groups, participants were instructed to write a half-page long essay on the given material for 25 min. The SW group was instructed to summarize the given text, while the AW group was instructed to create an argument based on what they learned. Specifically, the SW group was told to write more than three paragraphs, the total length of over a half-page. The group had to summarize various stages in youth’s cognitive development of music, which was mainly handled in the learning material. On the other hand, the AW group was instructed to write an argumentative essay. The length of the writing required was identical to the SW group. The task required the participants to pretend they were elementary school music teachers and propose a music class based on the cognitive development theories introduced in the material. Four developmental theorists of music were suggested by Zimmerman, Hargreaves, Gardner, and Swanwick & Tillman, each theory having distinctive views [21]. Students had to choose one of the theories and argue what they chose was better than the others. Following the study session, participants were asked to solve the Remote Associates Test, which also served as a filler task, for 15 min. Finally, they were given 20 min to complete a final test on the learning material.

### Remote associates test (RAT)

Remote Associates Test (RAT) is a test commonly used to assesses creativity [22]. This goes in context with one of the cognitive processes of higher-order thinking- creation or creative thinking [23]. To see how creative thinking is related to writing and final performance, fifteen questions were selected and used from the question pool published by Mednick [24, 25]. The student being tested has to think of a fourth word that is somehow related to the three words given and all questions have one definite answer.

### Measurement of academic performance

Final test questions were comprised of the rote-memory type and transfer type items. The ten rote-memory type items asked direct factual information on the given material and were worth 13 points. There were four transfer type items, which required the students to think a step further and apply what they learned to new situations. These items required not only an overall comprehension of the given material but also an application of it to different situations, which were worth 16 points. Thus, the maximum score of students could achieve was 29 points. Although most of the questions required the participants to write a short or narrative answer, these items had definite answers and guidelines for evaluating students’ performance. Furthermore, to rule out any subjective evaluation by the experimenters, three raters’ agreement for the scores on transfer type items was measured using Intraclass correlation. The coefficient value showed high agreement among the three raters (ICC (3, *k*) = .930).

### Writing scores

To measure participants’ writing performance, we scored their essays based on the criteria proposed by Lumley [26]. Specifically, we scored students’ writing on three criteria. First, we rated whether the writing was cohesive and organized (3 points). Second, whether it was appropriate to the task and given materials (2 points), in addition to whether the writing was easily comprehendible (2 points). Third, whether all aspects of presentation conventions including spelling, punctuation, and structure are handled skillfully (3 points). In total, the maximum writing score was 10 points.

### Statistical analysis

To examine the effect of different study conditions on academic performance, analysis of covariance (ANCOVA), linear regression analysis, and correlation analysis were performed. All statistical analyses were performed using SPSS 23 software (SPSS, Chicago, L, USA) and R (3.6.2. version; R Foundation, Vienna, Austria). The statistical significance for all tests was set as α < 0.05.

## Results

To begin with, ANCOVA was conducted to compare the academic performance of the students in the three groups. To summarize the results shown in Table 1, there was a significant difference in total test scores between the three groups (*P* = 0.002, η_p_^2^ = 0.102). The difference in scores for transfer type items was also significant (*P* < 0.001, η_p_^2^ = 0.249). However, no difference was found in the scores for rote-memory type items (*P* = 0.899, η_p_^2^ = 0.002).

**Table 1:** Academic performance by group and type of final test items

**Group**	SS (*n* = 46)	EW (*n* = 35)	AW (*n* = 35)
Total score (29 points)	13.34 (4.54)	17.11 (5.51)	16.54 (4.80)
Rote-memory items (13 points)	9.26 (1.97)	9.14 (2.40)	9.57 (1.70)
Transfer type items (16 points)	4.09 (2.03)	7.97 (3.73)	6.97 (3.00)
**Type of items**	*F*	*P*	η_p_^2^
Total score (29 points)	6.351	.002	.102
Rote-memory items (13 points)	0.106	.899	.002
Transfer type items (16 points)	18.616	<.001	.249

*F* (also known as value of F-distribution) describe the probability distribution, notably in the analysis of variance. *P* (also known as *p*-value) means statistical significance in the probability. η_p_^2^ (partial eta-score) is an effect size that is measure of the magnitude of a phenomenon in statistics. Data are shown as mean (standard deviation). *SS* Self-study, *SW* Summary writing, *AW* Argumentative writing. For each group, total scores, rote-memory item scores and transfer type item scores are given. Gender and age were adjusted

Analyzing the differences in more detail, the two writing groups showed significantly higher total test scores than the SS group (*P* = 0.007, η_p_^2^ = 0.064). The SS group scored significantly lower than the SW group (13.34 vs. 17.11, *P* = 0.003, η_p_^2^ = 0.077); however, no significant difference was found between the SS group and the AW group (13.34 vs. 16.54, *P* = 0.109, η_p_^2^ = 0.023). The average total score of the AW group was not significantly different from that of the SW group (16.54 vs. 17.11, *P* = 0.197, η_p_^2^ = 0.015).

For rote-memory type items, there was no significant difference between the three groups. The performance of the writing groups was not significantly higher than that of the SS group (*P* = 0.937, η_p_^2^ = 0.000). To re-emphasize, the score of the SS group for rote-memory type items was not significantly different from those of the SW group or the AW group (9.26 vs. 9.14, *P* = 0.858, η_p_^2^ = 0.000; 9.26 vs. 9.57, *P* = 0.756, η_p_^2^ = 0.001). The performance of the AW group was not significantly different from that of the SW group either(9.14 vs. 9.57, *P* = 0.648, η_p_^2^ = 0.002).

Regarding transfer type items, the writing groups performed significantly better than the SS group (*P* < 0.001, η_p_^2^ = 0.155). The SS group scored significantly lower than the SW group (4.09 vs. 7.97, *P* < 0.001, η_p_^2^ = 0.171), and the AW group (4.09 vs. 6.97, *P* = 0.006, η_p_^2^ = 0.067). However, the scores of the AW group and the SW group were not significantly different (6. 97 vs. 7.97, *P* = 0.077, η_p_^2^ = 0.028) (Table 2).

**Table 2:** Comparison of total scores, rote-memory item scores, and transfer type item scores between groups

Contrast	df	SS	*F*	*P*	η_p_^2^
Total scores					
Writing vs. SS	1	181.849	7.592	.007	.064
SS vs. SW	1	221.086	9.230	.003	.077
SW vs. AW	1	40.402	1.687	.197	.015
AW vs. SS	1	62.599	2.613	.109	.023
Rote-memory type item scores					
Writing vs. SS	1	.025	.006	.937	<.001
SS vs. SW	1	.129	.032	.858	<.001
SW vs. AW	1	.835	.209	.648	.002
AW vs. SS	1	.386	.097	.756	.001
Transfer type item scores					
Writing vs. SS	1	262.310	20.342	<.001	.155
SS vs. SW	1	295.550	22.919	<.001	.171
SW vs. AW	1	40.972	3.177	.077	.028
AW vs. SS	1	103.261	8.008	.006	.067

*F* (also known as value of F-distribution) describe the probability distribution, notably in the analysis of variance. *P* (also known as p-value) means statistical significance in the probability. η_p_^2^ (partial eta-score) is an effect size that measures the magnitude of a phenomenon in statistics. df is an abbreviation of degree of freedom. *SS* Self-Study, *SW* Summary writing, *AW* Argumentative writing. For each group, total scores, rote-memory item scores and transfer type item scores are given. Gender and age were adjusted

Data from the RAT task and the writing scores from participants in the writing groups were further analyzed. Correlation analysis for RAT scores for all three groups did show a weak positive correlation with performance in rote-memory items (*r(*114) = 0.18, *P* = 0.045). However, for participants in the writing groups, RAT scores did not show any significant correlation with the main study variables as demonstrated in Table 3.

**Table 3:** Correlations between main study variables

Variables	Writing scores	RAT scores	Total scores	Transfer type item scores	Rote-memory type item scores
Writing scores	–	–	–	–	–
RAT scores	0.14	–	–	–	–
Total scores	0.35^**^	0.16	–	–	–
Transfer type item scores	0.27^*^	0.10	0.91^***^	–	–
Rote-memory type item scores	0.34^**^	0.20	0.74^***^	0.41^***^	–

^*^P < 0.05, ^**^P < 0.01, ^***^P < 0.001

Lastly, the correlation between participants’ writing scores and their final performance was measured. Higher writing scores or performance should reflect higher levels of participation in learning. As a result, the writing scores of participants for all writing conditions combined showed a weak positive correlation with both performances in rote-memory (*r*(68) = 0.34, *P* = 0.003) and transfer type items (*r(*68) = 0.27, *P* = 0.022). However, linear regression analyses controlled for age and gender variables indicated that the writing scores significantly predicted performance on transfer type items for participants only in the AW group (*R*^2^ = 0.26, *F*(3, 31) = 5.123, *P* = 0.001) (Table 4).

**Table 4:** Multiple regression analysis of writing scores and performance on transfer type items

Independent variables	β	SE	*t*	*P*	95% CI	
					Upper	Lower
Summary writing (SW) group						
Age	−1.82	0.63	−2.92	0.007**	−3.099	−0.548
Gender	1.16	1.26	0.92	0.363	−1.40	3.719
Writing scores	0.29	0.35	0.91	0.372	−0.357	0.927
Constant	39.41	12.46	3.16	0.003**	14.0	64.826
Argumentative writing (AW) group						
Age	1.52	1.31	1.16	0.256	−1.153	4.184
Gender	1.06	1.04	1.02	0.316	−1.065	3.19
Writing scores	1.15	0.31	3.72	0.001***	0.521	1.787
Constant	−32.65	25.6	−1.28	0.211	−84.856	19.554

β is a dimensional parameter vector that is known as effects or regression coefficient. SE is an abbreviation of standard error. *t* (also known as *t*-statistic) is abbreviated from hypothesis test statistic. *P* (also known as *p*-value) means statistical significance in the probability. ^*^P < 0.05, ^**^P < 0.01, ^***^P < 0.001

## Discussion

Memorizing a large amount of knowledge still plays an important role in medical education. However, simply memorizing the given facts cannot foster students’ ability to grow as successful health professionals [27]. They need to train higher-order thinking skills to apply what they learned to novel situations as they cannot merely rely on memorization when dealing with various patients with different symptoms. This is why we investigated the effect of writing, which promotes higher-order thinking. By dividing the participants into three groups, those who study by themselves (SS), those who study by writing a summary text (SW), and those who study by writing an argumentative essay (AW), we tested each group’s performance on both rote-memory and the transfer of knowledge. While replicating previous literature on the effect of writing on students’ memory, our study has focused on finding empirical evidence that writing fosters the transfer of knowledge and higher-order thinking skills as well.

Following the first hypothesis that there would be no difference between groups in rote-memory scores, participants who learned through writing did not show significantly higher performance in rote-memory type items. These results are consistent with the previous research by Spirgel and Delaney, which reported that summary writing was not any more effective than restudying the text in solving short answer and multiple-choice questions [12]. Since the SS group learned the given material most similarly to re-studying the text, the fact that the SS group and the two writing groups performed similarly in rote-memory type items replicates previous works.

Expanding previous research on the effect of writing on learning, students were tested on their transfer of knowledge as well as how well they remember the information.  Following the second hypothesis, participants who learned through writing showed significantly higher performance in transfer type items than those who engaged in self-studying. Compared to students who just read the given material to comprehend and memorize the information, students who were involved in the writing task had to analyze what they had read and present what they had learned in their own words. Therefore, writing which requires higher-order thinking skills, such as analysis and evaluation, leads to a deeper level of learning [18] and a higher level of transfer of knowledge.

In addition to measuring the transfer of knowledge, two different writing tasks – summary and argumentative writing – were introduced in the experiment to test whether they have different effects on students’ learning. However, unlike the expectations, there were no differences between the two writing conditions, and the SW and the AW group showed similar performance in transfer type items. This pattern may be due to some characteristics of the writing task or our participant group. To elaborate, the summary writing task required participants in the SW group to delineate and summarize all four theories introduced in the given text. On the other hand, in the argumentative writing task, participants could write their claim by focusing on only one of the theories they preferred. Therefore, the SW group may have had an advantage over the AW group in answering the final test questions that dealt with the whole text. Next, the scores between the two writing groups may not have shown any difference due to the specificity of medical students. Medical students may already be familiar with writing summaries as they must have had memorized a large amount of knowledge in school. Conversely, students may lack experience in writing an argumentative essay and have weaker argumentation skills.

**Fig. 1:**
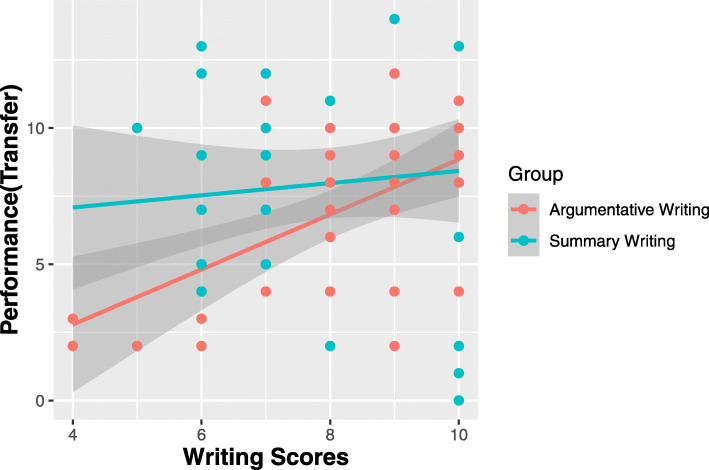
Linear Regression between writing scores and academic performance. Regression results representing how writing scores (0–10) affect students’ academic performance on transfer type items(0–16) for the two writing groups. Red points indicating summary writing(SW) group; blue points indicating argumentative writing(AW) group. For participants in SW groups, no clear pattern was found between performance and writing scores. On the other hand, participants in the AW group showed a linear relationship between transfer type item scores and writing scores

Results from further linear regression analysis imply similar conclusions (Table 4). Writing scores of both the SW and the AW group showed a weak positive correlation with performance in rote-memory and transfer type items (Table 3). However, linear regression results showed some different patterns on the effect of writing for the two writing groups (Fig. 1). While participants in the SW group achieved similar scores on transfer type items regardless of their writing scores, the test performance of participants in the AW group increased according to their writing scores. Since writing scores show a significant linear relationship with performance in transfer type items only in the AW condition, participants with poor writing skills in this group may not have benefited enough from the writing task. This also implies students who did not actively participate in the argumentative writing task may have lowered the average of the group performance, regardless of how argumentative writing promotes deeper learning than summary writing. Meanwhile, no significant relationship between writing and performance in transfer type items was found in the SW group, which suggests there was little individual variance in the learning effect of summary writing. Such different dynamics between the two different types of writing could have resulted in lower test scores in the AW group, or higher test scores in the SW group, bringing academic performance within the two writing groups to a similar level.

Last but not least, further analyses between RAT scores and the main study variables were performed to see whether creativity, or creative thinking which falls under higher-order thinking, is related to writing and final performance. RAT scores were only positively correlated with scores for rote-memory type items. This may be attributed to the fact that RAT focuses on measuring convergent thinking abilities, and not divergent thinking [28]. Creativity mainly consists of convergent thinking and divergent thinking; however, these two concepts are different. While convergent thinking skills involve the production of a single predetermined solution to a given problem like RAT, divergent thinking skills require the exploration of multiple possible solutions to generate creative ideas. In this sense, transfer type items that require the application of knowledge in various contexts may be closely related to divergent thinking than convergent thinking. The different focus of each assessment tool could have been the reason why the RAT did not show any significant correlations with the writing scores or performance in transfer type items.

To sum up, we were able to find some empirical evidence that writing can be a useful learning activity for medical students. First, we were able to replicate previous research by showing those who studied through writing performed similarly with those who self-studied the text in rote-memory items. Second, we went further to show writing has a significant influence on the level of transfer of knowledge for both summary and argumentative tasks, although no significant difference was found between the two conditions.

This study, however, still has some limitations. First, overall performance scores showed a floor effect. Specifically for transfer type items, the mean score of the three groups ranged from 4 to 7 points, much below the maximum of 16 points. Such an effect could have been due to the unfamiliar subject matter or high difficulty level of the items, which were selected from the National teacher certification examination in Korea. Nevertheless, the writing groups achieved significantly higher scores in transfer type items compared to the SS group. For future studies, it would be meaningful to replicate our results with a larger sample size and materials more closely related to what medical students study within the curriculum. Also, a more relevant measure of cognitive processes involved with higher-order thinking must be considered. The task used in our study was the RAT, which assesses creativity, specifically convergent thinking skills. Since almost no significant correlations were found between RAT scores and the main study variables, implementing and explaining other measurements will help us better understand the cognitive processes behind the effect of writing on learning. Lastly, due to time constraints, we could not fully investigate the effect of writing on academic performance in the long run. As we have seen from the linear regression results, students with weaker argumentation skills may not have fully benefited from the writing activity. Thus, if we help students increase their argumentation skills and practice argumentative writing over several periods, the benefits of writing on learning may turn out to be bigger. In this sense, it would be worthy of investigating whether the AW group shows better performance than the SW group after more training, and experiment with what other long-term benefits writing can bring to the medical students.

The current study provided new evidence to encourage the use of writing to complement the traditional ways of teaching in the medical education curriculum. Teachers are therefore encouraged to utilize writing in the classroom to help students develop thinking and apply what they learned to novel situations.

## Conclusion

Writing could be used as a useful learning tool that promotes higher-order thinking. As students have to analyze the given information and evaluate it to express their ideas in a compact piece of writing, higher-order thinking is promoted throughout the process. Therefore, our findings provide empirical evidence for writing to be adopted in medical classroom settings for greater benefits, in light of the transfer of knowledge. Overall, students who learned through writing showed a similar level of memorization with those who engaged in self-studying but higher performance in transfer type items. However, no particular difference in performance between SW and AW group was found, suggesting future studies are needed to identify what specific writing activities are helpful for medical school students. All in all, by actively using writing assignments in class, we expect medical students to acquire knowledge and foster higher-order thinking skills at the same time.

## Data Availability

The datasets used and/or analyzed during the current study are available from the corresponding author on reasonable request.

## Abbreviations

SS: Self-study group
SW: Summary writing group
AW: Argumentative writing group
RAT: Remote Associates Test
